# First record of White-eared Kob (*Kobuskobleucotis*) in Omo National Park, Ethiopia (Artiodactyla, Bovidae)

**DOI:** 10.3897/BDJ.10.e94114

**Published:** 2022-12-13

**Authors:** Tsyon Asfaw, Mihret Ewnetu, Abebayehu Moges, Assegid Gebre, Fikirte Gebresenbet, Hans Bauer

**Affiliations:** 1 Hawassa University, Wondo Genet College of Forestry and Natural Resources, Wondo Genet, Ethiopia Hawassa University, Wondo Genet College of Forestry and Natural Resources Wondo Genet Ethiopia; 2 Evolutionary Ecology Group, Biology, University of Antwerp, Wilrijk, Belgium Evolutionary Ecology Group, Biology, University of Antwerp Wilrijk Belgium; 3 Ethiopian Wildlife Conservation Authority, Addis Ababa, Ethiopia Ethiopian Wildlife Conservation Authority Addis Ababa Ethiopia; 4 Department of Natural Resources and the Environment, University of New Hampshire, Durham, NH, United States of America Department of Natural Resources and the Environment, University of New Hampshire Durham, NH United States of America; 5 Wildlife Conservation Research Unit, Biology, University of Oxford, Oxford, United Kingdom Wildlife Conservation Research Unit, Biology, University of Oxford Oxford United Kingdom

**Keywords:** range extension, White-eared Kob, first record, Omo National Park

## Abstract

White-eared Kob, *Kobuskobleucotis*, Lichtenstein & Peters, 1853, is known to occur in the Gambela-Boma landscape in western Ethiopia and South Sudan. They live in herds and are generally found near water, in such places as plains, woodlands, swamps, and flood plains. We deployed 36 camera traps in Omo National Park and one of them took two images of a White-eared Kob. This is the first documentation from Omo National Park and showing that its range extends further to Omo than previously known and, therefore, the entire area (Gambella to Omo) can be considered as a range extension.

## Introduction

*Kobuskob* is a medium-sized antelope. Females are smaller in size without horns and shaded with reddish or yellowish-ochre with white underside, face, ears and hocks, while males turn darker from rich cinnamon rufous or pale yellowish-brown with age. Males have large ridged horns that curve backwards, forwards and tip up forming an “S” shape. Three subspecies of kob have been described so far; the white-eared kob (*K.k.leucotis*), Ugandan kob (*K.k.thomasi*) and western kob (*K.k.kob*) following their geographical variation (Kingdon 2015).

White-eared kob, *Kobuskobleucotis*, Lichtenstein & Peters, 1853, is known to occur between the Gambela-Boma landscape in Ethiopia and South Sudan (Marjan 2014, Kingdon 2015, Schapira et al. 2016). They live in herds and are generally found near water, in plains, woodlands, swamps and flood plains (Kingdon 2015). The white-eared kob is morphologically distinct from the western and Ugandan sub-species and is listed as Least Concern on the IUCN Red List of Threatened Species. Nonetheless, the threats on white-eared kob populations may be increasing as poaching and habitat loss are major threats in the area (IUCN SSC Antelope Specialist Group 2016). Some white-eared kob herds are resident in South Sudan and Ethiopia, but most join one of the most spectacular, but least-studied mass migrations in Africa across the Boma-Gambela ecosystem (Schapira et al. 2016). Although it has not been studied extensively, Marjan (2014) carried out aerial surveys and estimated a population size of 792,782 individuals. The two armed conflicts (1985-2005 and 2013-2018) across the geographic range of the subspecies put serious pressure on local conservation efforts and partly explain why the white-eared kob migration remains poorly known (Schapira et al. 2016, Morjan et al. 2018). The migration which covers a total of 860 km in a circuit between Boma and Gambella is one of the notable transboundary ungulate migrations and its ecological importance needs to be clearly understood (Kauffman et al. 2021). In 2021, we conducted a camera trap survey to estimate the distribution of large carnivores in Omo, Maze and Chebera Churchura National Parks following the Omo Valley. We documented all mammal species captured in the three national parks and in this short communication, we are providing a first sighting (camera-trap photos) of white-eared kob from Omo National Park.

## Materials and Methods

**Figure 1.**
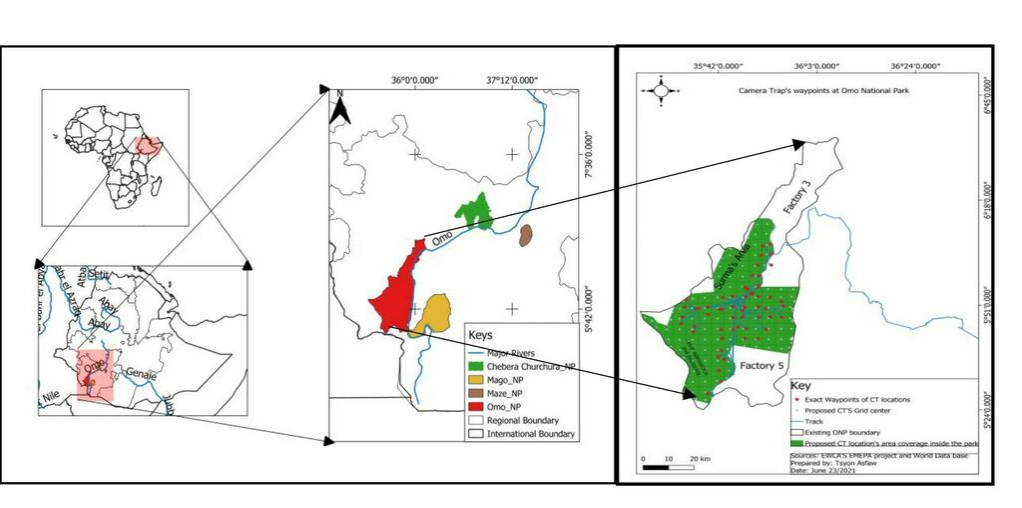
A map showing the location of ONP and the camera trap stations within ONP.

From 08 May 2021 to 03 August 2021, we put 53 camera traps (36 succesfully retrieved) for 2572 camera-trap nights across Omo National Park (ONP) overlayed in a 25 km^2^ grid. The mean distance between camera-trap stations was 4.26 km (1.34 km SD) (Fig. 1). ONP has low accessible roads, so most of the camera traps were set by foot and some areas had to be avoided due to security issues or proximity to people.

ONP is located in the lower Omo Valley in the Southern Nations, Nationalities and Peoples' Region (SNNPR), Ethiopia and has an area of 4068 km^2^ (Fig. 1). The average annual rainfall is 810 mm and the minimum and maximum temperatures range from 20°C to 40°C, respectively. The vegetation composition of the area includes savannah grassland, riparian forest and deciduous woodlands (Armaw and Molla 2022, Fig. 2).

Wildlife in ONP are threatened by different interacting threats including settlement, poaching, sugar cane processing industry inside Omo National Parks and a dam development project (Enawgaw et al. 2011, Gil-Romera et al. 2011, The Oakland Institute 2019).

## Results and Discussion

**Figure 3.**
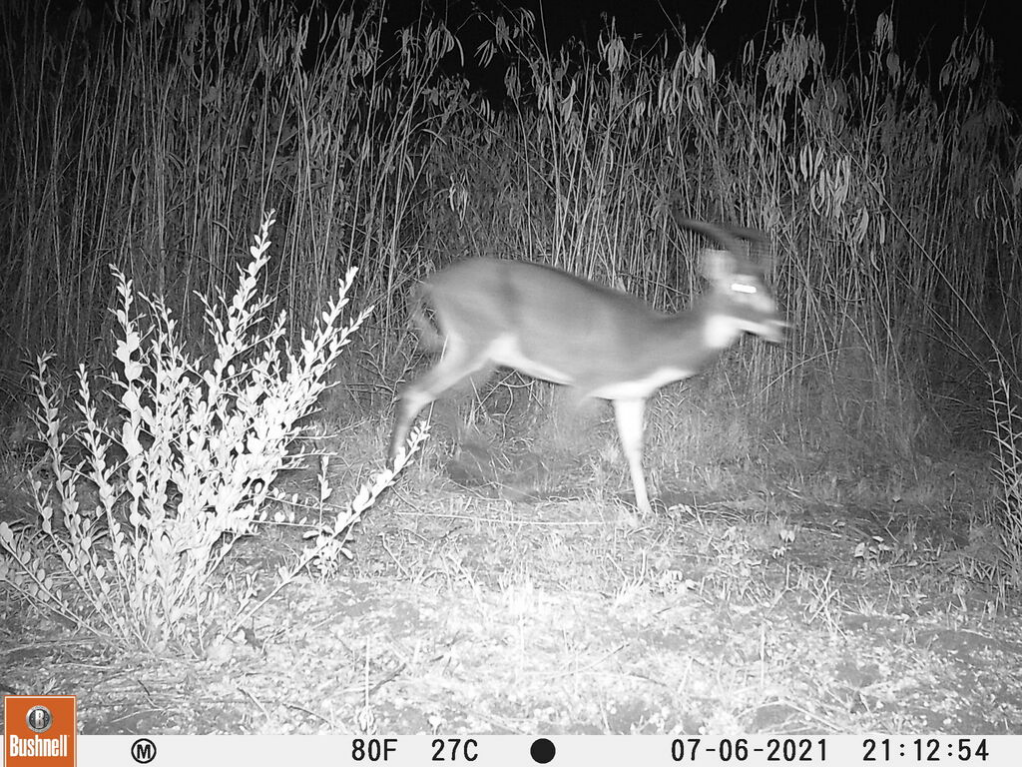
Camera trap image of white eared kob in ONP.

From the 2572 camera trap nights, we obtained 41 mammal species detections. All but one of these detections are of species that had already been recorded from ONP, including all five large carnivores of Africa. One of our cameras at 35.93084/5.84523 lat/lon captured a male white-eared kob (Fig. 3). We found no published evidence of white-eared kob outside the Gambela region of Ethiopia. The characteristic dark coat, white throat patch, white underside, face, ears and hocks are clearly seen in the photograph. The white-eared kob has not been previously recorded from ONP and is not listed in the park management plan (https://ethiopias-elephants.com/omo-national-park/). However, a personal observation by one of the authors (AG 2015, unpublished) reported a white-eared kob kill by local hunters in the nearby village of Berka in 2015. The author described the incident as something peculiar because locals were perplexed by the morphology of the animal and refused to eat the meat.

The habitat between Boma-Gambella and in ONP is largely intact and we speculate that the Boma-Gambella landscape is where these photographed white-eared kob came from (Schapira et al. 2016, IUCN SSC Antelope Specialist Group 2016). We propose, therefore, that the range of the subspecies be extended to include Omo and recommend that further studies be conducted across the wider landscape to establish the true extent of this subspecies.

## Figures and Tables

**Figure 2.**
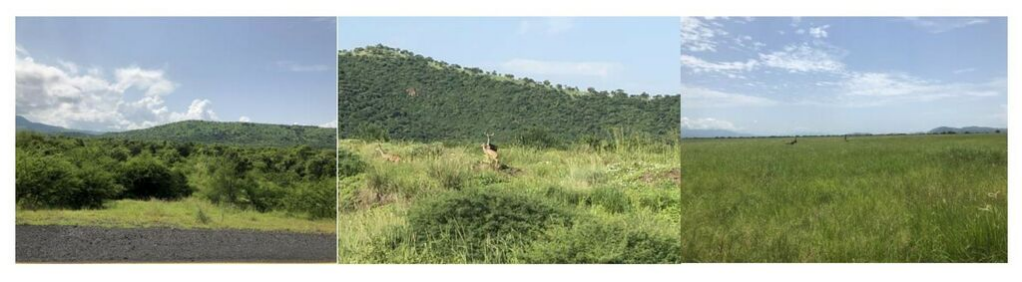
Common landscapes of ONP.
